# Spatial variation of healthcare resource use in Wales following the COVID-19 pandemic

**DOI:** 10.23889/ijpds.v9i5.2508

**Published:** 2024-09-18

**Authors:** Timothy Osborne, Rowena Bailey, Amy Mizen, Richard Fry

**Affiliations:** 1 Swansea University

## Background

The COVID-19 pandemic caused significant disruption to healthcare services and changed patterns of healthcare resource utilisation in Wales, but the geographic variation of this disruption is not known.

## Objective/Approach

We aimed to examine geo-spatial variation in the impact of the pandemic on healthcare service use, and identify socio-demographic and environmental factors associated with the geographical differences.

We accessed and linked individual electronic healthcare records within the Secure Anonymised Information Linkage (SAIL) Databank.

We calculated age-sex standardised rates of interactions across 1909 small areas in Wales, from January 2017 to December 2022. We compared rates in the 3 years pre-COVID (2017-2019) to the following 3 years (2020-2022) and investigated if spatial autocorrelation of results existed, using the Moran’s I test.

We linked area-level characteristics and carried out Geographically Weighted Regression (GWR) analysis to understand the socio-demographic and environmental characteristics associated with ‘hot’ and ‘cold’ spots of healthcare resource use.

## Results

We included 1,277,532 individuals with 7,502,485 healthcare service interactions related to a long-term condition. 

Across the 1909 small areas in Wales, we observed large variations in age-sex standardised rates of healthcare interactions. Area-level general practice and hospital admission rates decreased by a median of 22%, and 13.1% respectively, from 2017-2019 to 2020-2022. The change in general practice rates were more significantly spatially autocorrelated than the change in hospital admission rates.

Spatially varying relationships between healthcare resource use and area-level characteristics such as income deprivation, travel time to general practice/hospital, have been analysed using GWR (results to be finalised).

## Conclusions/Implications

We have found that the COVID-19 pandemic impacted population level healthcare resource use in some areas of Wales more than others. Identifying the underlying characteristics causing these differences will help inform policy to provide targeted services to areas and subgroups of the population.

